# Development and Validation of a Reporter-Cell-Line-Based Bioassay for Therapeutic Soluble gp130-Fc

**DOI:** 10.3390/molecules24213845

**Published:** 2019-10-25

**Authors:** Lei Yu, Chuncui Jia, Wenrong Yao, Dening Pei, Xi Qin, Chunming Rao, Junzhi Wang

**Affiliations:** Division of Recombinant Biological Products, National Institutes for Food and Drug Control, Beijing 100050, China; yulei@nifdc.org.cn (L.Y.); chuncui319@163.com (C.J.); yz1322@126.com (W.Y.); peidening@nifdc.org.cn (D.P.); Qinxi@nifdc.org.cn (X.Q.)

**Keywords:** sgp130-Fc, reporter assay, digital PCR

## Abstract

Soluble glycoprotein 130 kDa (sgp130)-Fc fusion protein, an innovative therapeutic bio-macromolecular drug specifically targeting IL-6 trans-signaling, proved to have good potential for application in the treatment of chronic inflammatory diseases. A simple and quick bioassay for sgp130-Fc was developed in this study. First, a stable reporter cell line was obtained by transfecting CHO-K1 cells with a sis-inducible element (SIE)-driving luciferase reporter gene (CHO/SIE-Luc). Sgp130-Fc could inhibit the expression of luciferase induced by IL-6/sIL-6Rα complex, and the dose–response curve fitted the four-parameter logistic model, with 50% inhibitive concentration (IC_50_) being about 500 ng/mL and detection range between 40 and 5000 ng/mL. Both the intra-assay and inter-assay coefficient of variation (CV) were below 10.0%, and the accuracy estimates ranged from 94.1% to 106.2%. The assay indicated a good linearity (*R²* = 0.99) in the range of 50% to 150% of optimized initial concentration. No significant difference was found between the test results of new assay and BAF3/gp130 proliferation assay (unpaired *t* test, *p* = 0.4960, *n* = 6). The dose-response effect and copy number of the luciferase gene was basically unchanged after long-term culture (up to passage 60), demonstrating the stability of CHO/SIE-Luc cells. These results suggested that the new reporter assay was suited to routine potency determination of therapeutic sgp130-Fc.

## 1. Introduction

An extensive body of research has demonstrated interleukin-6 (IL-6) is centrally involved in the development of chronic inflammatory diseases [1,2,3,4]. Furthermore, it also plays a role in the development of cardiovascular diseases and cancers [5,6,7]. These broad and multiple functions of IL-6 make it a promising therapeutic target [7,8,9,10]. IL-6 can signal through distinct pathways on target cells. On hepatocytes and leukocytes, IL-6 initially binds to its membrane-bound IL-6 receptor α-subunit (IL-6Rα), and then recruits the signal transducing β-subunit, glycoprotein 130 kDa (gp130), forming a hexameric signaling complex, which was termed “classic signaling”. Cells that do not express IL-6Rα, but only the ubiquitously expressed gp130 on the surface can still be responsive to IL-6 through an alternative process termed “IL-6 trans-signaling”, which is mediated by soluble IL-6Rα (sIL-6Rα), formed by limited proteolysis of membrane-bound IL-6Rα or alternative mRNA splicing [11,12,13,14]. Many studies proved that IL-6 trans-signaling via sIL-6Rα played a critical role in chronic inflammation and cancer, which is a new target for therapeutic bio-drugs [15,16,17]. The soluble ectodomain of gp130 (sgp130) could specifically inhibit IL-6 trans-signaling but not interfere with classic signaling [18,19]. Endogenous sgp130 was considered to be a natural antagonist of the IL-6/sIL-6Rα complex in vivo, probably to block IL-6 trans-signaling during inflammatory diseases. Two sgp130 linked by a Fc portion of human IgG1 (sgp130Fc) was shown to be 10–100 times more effective than sgp130 alone [19,20]. Furthermore, sgp130-Fc also inhibited IL-6 trans-signaling exclusively and has no affinity to IL-6 or IL-6Rα alone, suggesting that it has no effect on classic signaling and produces less adverse reactions compared with other IL-6 antagonists [20,21,22]. A recombinant sgp130-Fc product is currently in a phase II clinical trial for treatment of chronic inflammatory diseases (Clinicaltrials.gov) [23,24].

Biologically active recombinant proteins, especially fusion proteins with high molecular weight, cannot be fully characterized by physicochemical methods alone. Thus, biological assays (bioassays) are important for their biological characterization and potency determinations [25,26]. Activity evaluation is critical in the research and development (R and D) stage of recombinant protein products, as well as quality control of the production process and final products [27,28]. Currently, the in vitro activity of sgp130-Fc is mainly determined by its ability to inhibit the proliferation of BAF3/gp130 cells stimulated by the IL-6/sIL-6Rα complex [18,29]. In this study, we developed a reporter assay for sgp130-Fc based on its intracellular signal pathway. It was confirmed that the signal transducer and activator of transcription-3 (STAT3) plays a key role in the physiological function of IL-6, by binding and activating the sis-inducible element (SIE) site located in promoters of target genes [7,9,30]. IL6R^−^and gp130^+^ CHO-K1 cells were stably transfected with SIE-driving luciferase reporter plasmid, and the production of luciferase could be stimulated by the IL-6/sIL-6Rα complex, which was blocked by sgp130-Fc in a dose-dependent manner. The developed assay was fully optimized and validated, and the stability of reporter cells was also evaluated.

## 2. Results

### 2.1. Development of a Stable Reporter Cell Line for sgp130-Fc

**Figure 1:**
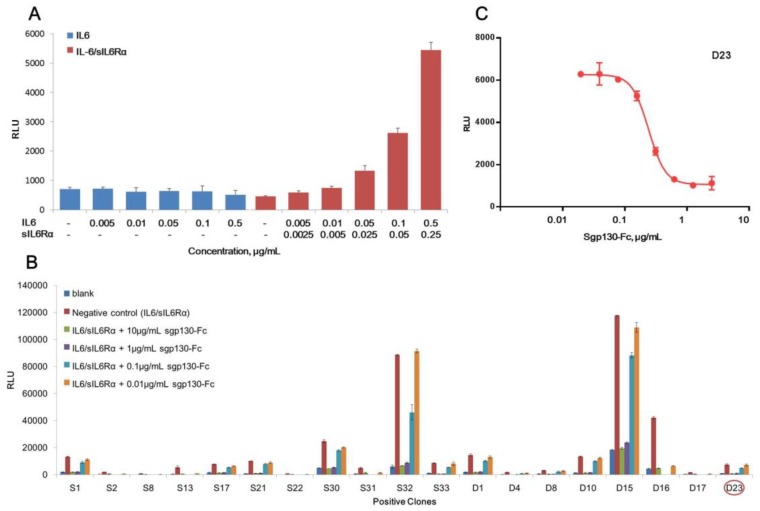
Development of a stable reporter cell line for sgp130-Fc. (**A**) Responsiveness of CHO-K1 transfected with sis-inducible element (SIE)-driving luciferase reporter to IL6 or IL6/sIL6Rα complex. (**B**) Responsiveness of different positive clones (clone screening). (**C**) Dose–response curve of D23 clone. RLU = relative luciferase units. Each plot represents the mean of three replicates.

Purified and endotoxin-free pGL4.47 [luc2p/SIE/Hygro] plasmid was transfected into CHO-K1 cells, and the responsiveness to IL-6 or IL6/sIL6Rα complex was tested by determination of luciferase activity. As shown in Figure 1A, only with the presence of both IL6 and sIL6Rα, the luciferase activity raised dose-dependently. IL-6 alone couldn’t induce the increase of luciferase, demonstrating that IL-6 classic-signaling pathway was inactive in CHO-K1 cells, and IL6/sIL6Rα complex-induced luciferase production was achieved specifically by the trans-signaling pathway. Nineteen positive clones responsive to the IL6/sIL6Rα complex obtained by hygromycin B selection were shown in Figure 1B. For all of them, the increase of luciferase activity induced by the IL6/sIL6Rα complex (0.5 μg/mL IL6 + 0.25 μg/mL sIL6Rα) could be blocked by sgp130-Fc in a dose-dependent manner. Although clones S32 and D15 exhibited stronger induction of luciferase, their background of luciferase was also higher, suggesting that more copies of luciferase genes were integrated. Considering that transgenic cells with high copies of exogenous genes are generally unstable [31,32,33], the clones with medium copies of luciferase are more suited to routine bioassay. Combining dose-response behavior and physiological status of cells, the D23 clone was finally chosen and designated as the CHO/SIE-Luc cell line. As shown in Figure 1C, the dose-response curve fitted the four-parameter logistic model and demonstrated good linearity (*R^2^* above 0.98). We next set out to develop a robust reporter assay for potency determination of sgp130-Fc using CHO/SIE-Luc. 

### 2.2. Method Development and Optimization

**Figure 2:**
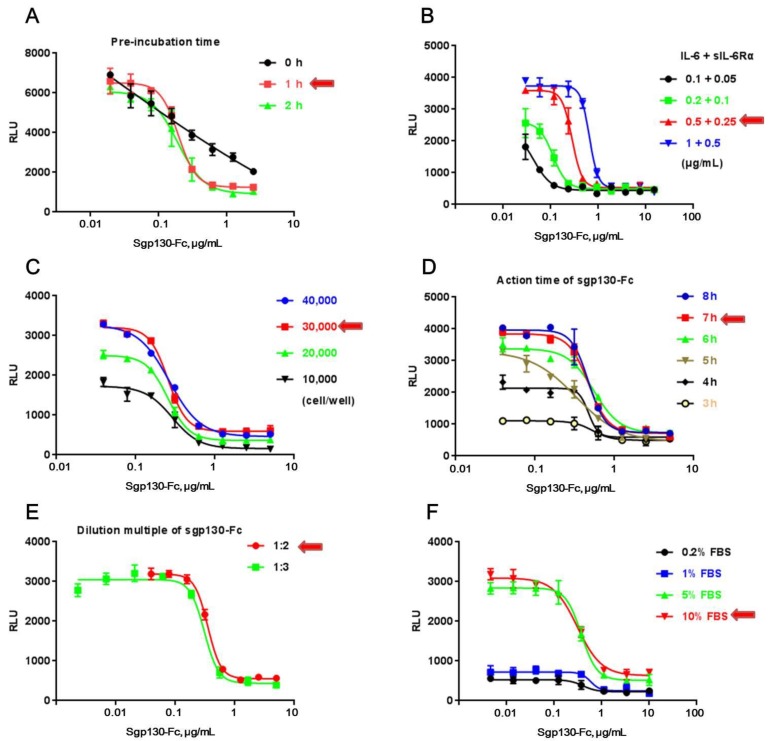
Optimization of the experiment parameters. (**A**) Pre-incubation time of sgp130-Fc with IL-6/sIL-6Rα complex. (**B**) IL-6/sIL-6Rα concentration. (**C**) Cell number per well. (**D**) Action time of sgp130-Fc. (**E**) Working concentration and dilution multiple of sgp130-Fc. (**F**) Working concentration of FBS in assay medium. The optimal experiment parameters are directed by red arrows. Each plot represents the mean of three replicates.

CHO/SIE-Luc was employed to develop a reporter assay for sgp130-Fc. Bioassay development proceeds through a series of experiments to identify those assay factors that support a reliable and robust bioassay qualified for routine use. In this study, those experiments were conducted one factor at a time (OFAT), studying each assay factor separately to identify ideal conditions, including pre-incubation time of sgp130-Fc with the IL-6/sIL-6Rα complex (Figure 2A), working concentration of IL-6 and sIL-6Rα (Figure 2B), cell number per well (Figure 2C), action time and detection range of sgp130-Fc (Figure 2D,E), and working concentration of FBS in assay medium (Figure 2F). In each test, except the assay factor to be studied, all other experiment parameters were identical among different test groups (according to Section 2.3).

It’s worth noting that pre-incubation of sgp130-Fc with the IL-6/sIL-6Rα complex at 37 °C played an important role in the assay (Figure 2A), which might help sgp130-Fc block the action of the IL-6/sIL-6Rα complex more effectively. Furthermore, only with a higher concentration of FBS could the IL-6/sIL-6Rα complex induce the production of luciferase effectively, suggesting that some accessory factors in FBS could assist the action of the IL-6/sIL-6Rα complex. Table 1 summarized the optimal conditions used in all subsequent experiments. With the optimal condition, 50% inhibition concentration (IC_50_) was about 500 ng/mL and detection range was between 40 and 5000 ng/mL. 

### 2.3. Method Validation

To validate the developed CHO/SIE-Luc based reporter assay, an in-house reference for sgp130-Fc was used, and its potency was defined as 100% in subsequent tests. Sgp130-Fc samples were tested in triplicates for each dose, and their respective potencies were estimated using the in-house reference as a reference for activity. All tests were conducted according to International Council for Harmonisation of Technical Requirements for Pharmaceuticals for Human Use (ICH) Guidelines Q2, including specificity, precision, accuracy, and linearity. In addition, the consistency with BAF3/gp130 proliferation assay in test results was also evaluated.

#### 2.3.1. Specificity

**Figure 3:**
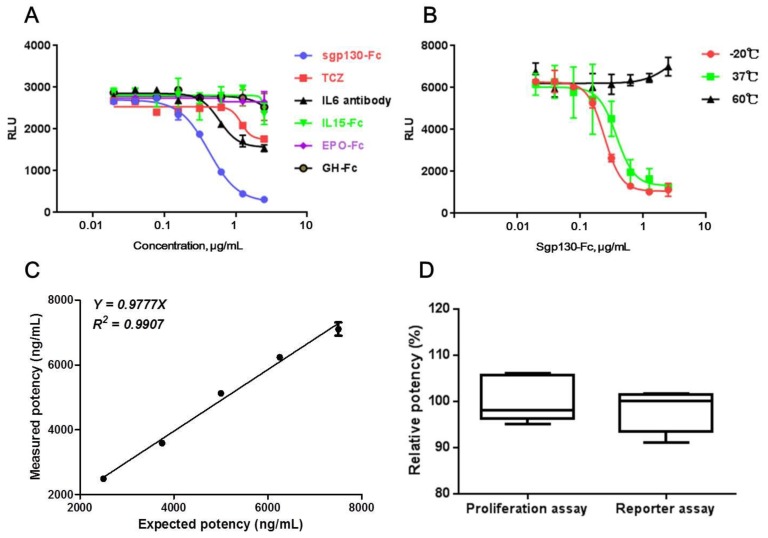
Method validation. (**A**) Reactivity of CHO/SIE-Luc cells to IL6 antibody, IL6R antibody (TCZ), and other Fc-fused protein drugs (IL15-Fc, EPO-Fc, and GH-Fc). (**B**) Reactivity of CHO/SIE-Luc cells to heat-inactivated sgp130-Fc proteins. (**C**) Linearity of CHO/SIE-Luc based reporter assay. (**D**) Comparison between the test results of BAF3/gp130 cell-based proliferation assay and CHO/SIE-Luc cell-based reporter assay (*n* = 6). Each plot represents the mean of three replicates in (**A**,**B**,**C**).

Specificity of a bioassay always refers to the capacity of the bioassay to distinguish between different but related biopharmaceutical molecules. For this purpose, other IL6-targeted proteins, including an anti-IL6 antibody and an anti-IL6R antibody (Tocilizumab, TCZ), and other Fc-fused therapeutic proteins, including IL15-Fc, Epo-Fc, and GH-Fc, were assessed to find out whether they could affect the production of luciferase induced by the IL-6/sIL-6Rα complex in CHO/SIE-Luc. As shown in Figure 3A, although anti-IL6 and anti-IL6R antibody had partial inhibition on luciferase production, the inhibiting effect was significantly weaker than sgp130-Fc. As for other Fc-fused proteins, no obvious effect on luciferase production was observed. 

The specificity of the bioassay was also assessed by the presence of degraded, aggregate, or other inactivated components of sgp130-Fc. It is known that, with an increasing temperature, proteins may undergo conformational changes, subsequently leading to degradation, aggregation, or other reactions [34]. Therefore, thermal inactivation of sgp130-Fc was induced by incubation at 37 or 60 °C for 24 h. Compared with the sample stored at −20 °C, the sample incubated at 37 °C showed attenuated activity, and, even worse, for the sample incubated at 60 °C, no obvious activity was observed, suggesting that the reporter assay could distinguish heat-inactivated sgp130-Fc molecules effectively (Figure 3B).

#### 2.3.2. Precision, Accuracy, and Linearity

*Precision.* In order to determine the precision of the new reporter assay, we ran the assay on three different days, with three repeated analyses of sgp130-Fc samples each day. Such a design made it possible to better understand the plate-to-plate variability, as well as the inter-assay variation. As listed in Table 2, our analyses resulted in intra-assay (within-day) and inter-assay (between-day) CV of IC_50_ values below 10%. No statistically significant day effect was found (Kruskal–Wallis test, *p* = 0.4298, *n* = 3).

*Accuracy.* According to the ICH Q2 (R1) PART II, the accuracy of a method should be reported as the rate of recovery of a known added amount of analyte in a sample, so we verified the percentage of a sgp130-Fc in-house reference recovered from sgp130-Fc test samples by conducting an analysis of six separate assays on different days. As shown in Table 3, the inter-assay CV was satisfactory, and the 95% confidence interval (CI) of the recovery rates was 97.8% to 107.7% (*n* = 6), demonstrating that no significant interference of matrix formulation was found.

*Linearity.* The linearity of an analytical method is its ability to give test results which are directly proportional to the concentration of analyte in the sample. For linearity validation, sgp130-Fc in-house reference at 5000 ng/mL and test samples at 2500, 3750, 5000, 6250, and 7500 ng/mL (50% to 150% of the optimized initial concentration) were prepared and tested by reporter assay. The test was performed repeatedly on three different days, and the obtained data were analyzed using a linear regression model. As shown in Figure 3C, the linearity plots for the measured potencies of test samples versus the expected potencies displayed a linear behavior. The slope of 0.9777 suggested high correlations between expected values and measured values, and *R^2^* of 0.99 showed good linearity in the range of 50% to 150% of the optimized initial concentration.

#### 2.3.3. Consistency with BAF3/gp130 Proliferation Assay in Test Results

To evaluate the consistency between CHO/SIE-Luc-based reporter assay and BAF3/gp130-based proliferation assay, the relative potencies of sgp130-Fc test samples were tested six times by two assays, respectively. Reporter assay was performed using optimized conditions, and BAF3/gp130-based proliferation assay was conducted according to “2.4 BAF3/gp130 proliferation assay”. The mean, 95% CI of mean, and standard deviation (SD) of test results were shown in Figure 3D. No statistically significant difference was found between the test results of two assays (unpaired *t* test, *p* = 0.4960, *n* = 6).

### 2.4. Stability of CHO/SIE-Luc Cell Line

As a genetically modified cell-line-based bioassay, the stability of cells guarantees the stability of the assay, including genetic and function stability, which should be evaluated. Function stability could be evaluated by dose–effect curves in response to test sample, and genetic stability could be evaluated by the copy number of modified gene, after long-term culture.

#### 2.4.1. Function Stability

**Figure 4:**
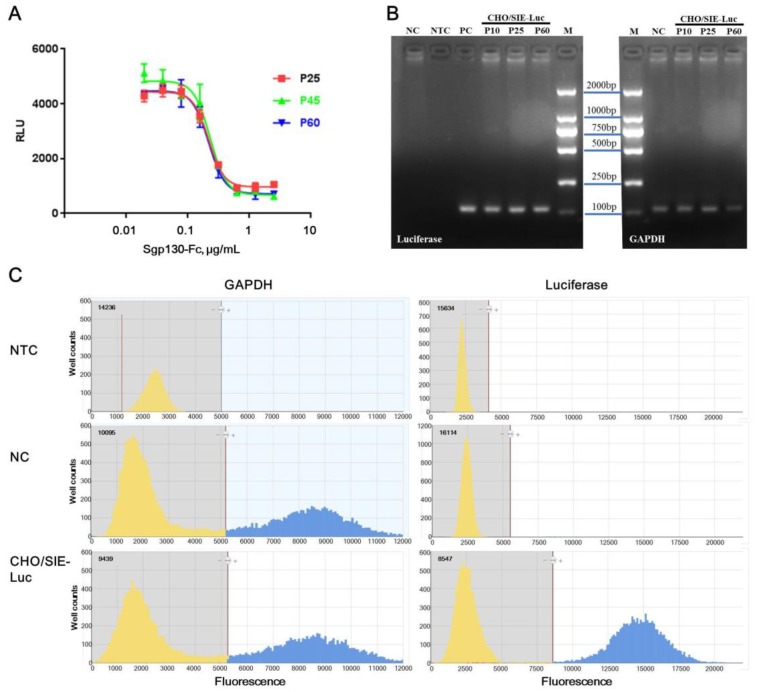
Function and genetic stability of CHO/SIE-Luc cells. (**A**) Dose-response curves of CHO/SIE-Luc cells at passage 25, 45, and 60. Each plot represents the mean of three replicates. (**B**) PCR products of luciferase and GAPDH. (**C**) Output of digital PCR. Yellow and blue area represents the pool of negative and positive reactions, respectively. NTC: no template control; NC: native CHO-K1; PC: positive control (SIE-diving luciferase reporter plasmid).

To evaluate the function stability of CHO/SIE-Luc cells to sgp130-Fc, the responsiveness of cells at passage 25, 45, and 60 were tested. Figure 4A showed that the cells at different stages behaved nearly indistinguishably. The IC_50_ was 217.1, 230.4, and 217.7 ng/mL, the slope of the curves were −3.5, −3.2 and, −3.3, and the SNR was 4.6, 7.2, and 6.2, for cells at passage 25, 45, and 60, respectively. Although a slight fluctuation existed, which is recognized to be common in cell-based assay, the responsiveness of CHO/SIE-Luc cells to sgp130-Fc was proved to be moderately stable between passage 25 and passage 60.

#### 2.4.2. Genetic Stability

To evaluate the genetic stability of CHO/SIE-Luc cells, the copy number of the luciferase gene in CHO/SIE-Luc cells was tested by digital PCR, an emerging analysis technology for absolute quantification of gene copies. Digital PCR works by partitioning a sample of DNA into many individual, parallel PCR reactions; some of these reactions contain the target molecule (positive), while others do not (negative) [35,36]. Although there’s generally no need for standards or endogenous controls for digital PCR, the accuracy of test results is still dependent on the quantification of template DNA. Considering the concentration of the extracted genome was determined by 260 nm absorbance, which was rough and inaccurate, GAPDH was still used as an internal control gene in our study. To confirm the specificity of primers and probes, real-time PCR was performed first, and PCR products were tested by electrophoresis. Target bands were recycled and verified by sequencing (Figure 4B). Combined test results of real-time PCR, DNA sequencing and digital PCR, the specificity of primers, and probes for luciferase and GAPDH was acceptable. The output of digital PCR for no template control (NTC), NC (native CHO-K1), and CHO/SIE-Luc was shown in Figure 4C. The copy numbers of luciferase and GAPDH in CHO/SIE-Luc cells at passage 10, 30, and 60 were listed in Table 4, and the relative copy numbers of luciferase (copies per copy GAPDH) were basically consistent. Therefore, the copy number of luciferase in CHO/SIE-Luc cells was proved to be highly stable between passage 10 and passage 60.

## 3. Discussion

Biological activity is a critical quality attribute (CQA) in the quality control of therapeutic recombinant proteins, and the development of a proper and stable bioassay is essential in research and development stage of innovative protein products [25,26,27,28]. Sgp130-Fc, a specific inhibitor of IL-6 trans-signaling, was proved to have good potential application in the treatment of chronic inflammatory diseases. For in vitro assay of sgp130-Fc, an IL6R^−^ and gp130^+^ cell line is necessary to avoid the interference of classic signaling. Native BAF3 cells are murine pre-B cells, which do not express IL-6, IL-6Rα, or gp130 and BAF3/gp130 cells were BAF3 cells stably transfected with human gp130 [37,38]. IL-6/sIL-6Rα complex could stimulate the proliferation of BAF3/gp130 cells, but it is not essential for the growth of BAF3/gp130 cells. To preserve the growth dependence on IL-6/sIL-6Rα complex, tedious work was needed in maintenance of BAF3/gp130 cells, such as low-density culture and routine monitoring. In this study, we developed a novel reporter assay using a CHO/SIE-Luc cell line, which was only responsive to IL-6/sIL-6Rα but not IL-6 alone, excluding the interference of IL-6 classic signaling. Sgp130-Fc could inhibit the increase of luciferase production induced by the IL-6/sIL-6Rα complex, and the dose–response curve fitted the four-parameter logistic model well, yielding a coefficient of determination of 0.98 or higher. Both intra-assay and inter-assay CV was below 10.0%, and the accuracy estimates ranged from 94.1% to 106.2%. The measured bioactivity versus expected bioactivity indicated a good linearity (*R²* = 0.99). No significant difference was found between the test results of new reporter assay and BAF3/gp130 proliferation assay, but compared with BAF3/gp130 proliferation assay, the operation of reporter assay was much simpler (only several steps) and quicker (completed within 24 h), and the maintenance of the CHO/SIE-Luc cell line did not need extra care. Furthermore, a chemiluminescence signal is superior to live cell dyes on sensitivity and exclusiveness, and this intracellular-signal-pathway-based bioassay has better specificity, avoiding the interference of other components with potential effects on cell proliferation in medium or serum. Considering that the stability of CHO/SIE-Luc cells is an important premise for the assay, both function stability and genetic stability were evaluated after long-term culture (up to passage 60). For function-stability evaluation, Figure 4A showed that the cells at different stages behaved nearly indistinguishably. For genetic stability evaluation, we developed a digital PCR-based assay. The adoption of the GAPDH gene as an internal control improved the repeatability of test results. The measured copy number of the luciferase gene in CHO/SIE-Luc cells at different stages was basically consistent (Table 4). Collectively, these results suggested that the new reporter gene assay was suited to routine potency determination of therapeutic sgp130-Fc. 

## 4. Materials and Methods

### 4.1. Cells and Materials

The CHO-K1 cell line was obtained from the American Type Culture Collection (Manassas, VA, USA) and maintained in F12K containing 10% fetal bovine serum (FBS). The BAF3/gp130 cell line was obtained from I-Mab Biopharma (Shanghai, China) and maintained in DMEM containing 5% FBS, 0.1 μg/mL IL-6, and 0.05 μg/mL sIL-6Rα. F12K, DMEM, FBS, and hygromycin B were purchased from Gibco (Grand Island, NY, USA). IL-6 and sIL-6Rα were purchased from PeproTech (Rocky Hill, NJ, USA). PGL4.47 (luc2p/sis-inducible element (SIE)/Hygro) firefly luciferase reporter plasmid, ViaFect™ transfection reagent, MTS reagent, and the Bright-Glo™ Luciferase Assay System were purchased from Promega (Madison, WI, USA). The TIANamp Genomic DNA Kit was purchased from TIANGEN (Beijing, China). The TaqMan Fast Advanced Master Mix, 3D Digital PCR Master Mix v2, and 3D Digital PCR 20K Chip Kit were purchased from ABI (Foster City, CA, USA). Sgp130-Fc, Tocilizumab (TCZ), IL15-Fc, Epo-Fc, and growth hormone (GH)-Fc were archived therapeutic drugs that were preserved at 4 or −80 °C in our laboratory. 

### 4.2. Preparation of a Stable Reporter Cell Line

SIE-driving luciferase reporter plasmid pGL4.47 was transfected into CHO-K1 cells, using ViaFect™ transfection reagent, according to the manufacturer’s instructions. The transfected cells received regular changes of selection medium (F12K medium supplemented with 10% FBS and 300 μg/mL of hygromycin B) and were continuously cultured for four weeks. Then, a clonal cell line derived from a single cell was produced by limiting dilution, and the specific approach was that the stably transfected cells were collected and adjusted to one cell per 200 μL in selection medium, and 100 μL per well was seeded to a 96-well plate. After isolating the clones, clone scale-up and screening assessments (responsive to IL-6/sIL-6Rα/sgp130-Fc) were performed. The desired cells were named CHO/SIE-Luc and maintained in F12K medium supplemented with 10% FBS and 300 μg/mL of hygromycin B.

### 4.3. Reporter Assay

CHO/SIE-Luc cells in analysis medium (F12K supplemented with 10% FBS) were seeded in 96-well white plate (3 × 104 per well in a total volume of 80 μL) and incubated at 37 °C in a CO_2_ incubator for 16–18 h. A mixture of 5 μg/mL IL-6 and 2.5 μg/mL sIL-6Rα in analysis medium was freshly prepared, and then mixed with the same volume of serially diluted sgp130-Fc samples or references, followed by incubation at 37 °C for 1 h. After that, 20 μL of the IL-6/ sIL-6Rα/ sgp130-Fc mixture was added to the cell plate, which was then incubated at 37 °C in a CO_2_ incubator for 7 h. Then, 100 μL of Bright-Glo™ Assay reagent was added, and the plate was subsequently shaken for 5 min on a titer-plate shaker. Luminescence values were finally determined by reading on a Chemiluminescence Microplate Reader (SpectraMax M5, Molecular Devices, San Jose, CA, USA). 

### 4.4. BAF3/gp130 Proliferation Assay

BAF3/gp130 cells in analysis medium (DMEM supplemented with 5% FBS) were seeded in a 96-well plate (5 × 10^3^ per well in a total volume of 90 μL) and incubated at 37 °C in a CO_2_ incubator for one hour. A mixture of 2 μg/mL of IL-6 and 1 μg/mL of sIL-6Rα in analysis medium was freshly prepared and added to the cell plate (5 μL per well). Then, 5 μL of diluted sgp130-Fc test samples or references was added, followed by incubation at 37 °C in a CO_2_ incubator for 72 h. After that, 20 μL of MTS was added, the plate was incubated at 37 °C in a CO_2_ incubator for 4 h, and then it was read at 490 nm on a SPECTRAmax plate reader. 

### 4.5. PCR Primers and Probes

Primers and probes were synthesized by Sangon Biotech (Shanghai, China). The sequences of forward primer, reverse primer, and probe for luciferase were 5′-GGCTGAATACAAACCATC-3′, 5′-CGTTGTAGATGTCGTTAG-3′, and 5′-(FAM) CACAGCCACACCGATGAACAG (TAMRA)-3, respectively. The sequences of forward primer, reverse primer, and probe for glyceraldehyde-3-phosphate dehydrogenase (GAPDH) were 5′-AAGGCTGAGAATGGAAAG-3′, 5′-CCAGTAGATTCCACAACA-3′, and 5′-(FAM) CATCACCATCTTCCAGGAGCGA (TAMRA)-3′, respectively.

### 4.6. Digital PCR

Genomic DNA of native CHO-K1 or CHO/SIE-Luc was prepared using a TIANamp Genomic DNA Kit according to the manufacturer’s instructions and then quantified by 260 nm absorption. Genomic DNA template for luciferase and GAPDH were diluted to 100 and 6.5 ng/mL, respectively. The total volume of the PCR reaction was 14.5 μL, composed of 1.175 μL nuclease-free water, 1.35 μL forward primer (10 µM), 1.35 μL reverse primer (10 µM), 0.375 μL probe (10 µM), 7.25 μL 3D Digital PCR Master Mix (2×), and 3 μL genomic DNA template (100 ng/mL for luciferase or 6.5 ng/mL for GAPDH). Nuclease-free water was used as no-template control. Load the total 14.5 µL of reaction per QuantStudio™ 3D Digital PCR Chip, and run PCR on a QuantStudio 3D Digital PCR System (Foster City, CA, USA). The PCR procedure consisted of three stages: 90 °C for 10 min, 39 cycles of 56 °C (luciferase)/54 °C (GAPDH) for 30 s, 60 °C for 2 min and 95 °C for 30 s, and 60 °C for 2 min. Data were analyzed using the QuantStudio 3D AnalysisSuite™ Cloud Software (version 3.1.6-PRC-build2, Thermo Fisher Scientific, Carlsbad, CA, USA). 

### 4.7. Data Analysis and Statistics

All of the statistical analyses were performed using SoftMaxPro (version 5.4.1, Molecular Devices, Sunnyvale, CA, USA) and GraphPad Prism 7.0 (GraphPad Software Inc., San Diego, CA, USA). The sigmoidal curve and the concentration for 50% maximal effect (IC_50_) were calculated through a four-parameter model (dose–response–stimulation). The relative potency of sgp130-Fc was shown as the ratio of the IC_50_ values of an in-house reference to the IC_50_ values of test samples. Comparisons between two groups were performed using an unpaired *t* test, and multiple comparisons were performed using a Kruskal–Wallis test. The *p*-values < 0.05 were deemed to be statistically significant.

## 5. Conclusions

In this study, a simple and time-saving bioassay was developed for the potency determination of sgp130-Fc. The proposed bioassay was proved to be precise, reproducible, and robust. It has the potential to be used for the routine analysis of bioactivity during the research, development, and manufacture of therapeutic sgp130-Fc. Our work also provides an important reference for the development of this type of bioassays for other therapeutic recombinant proteins.

## Figures and Tables

**Table 1:** Optimized experiment parameters of reporter assay for sgp130-Fc.

Experiment Parameters	Optimized Values
Pre-incubation time of sgp130-Fc with IL-6/sIL-6Rα	1 h
IL-6 and sIL-6Rα working concentration	0.5 μg/mL IL-6 and 0.25 μg/mL sIL-6Rα
Cell number (per well)	30,000
Action time of sgp130-Fc	7 h
FBS working concentration	10%
Detection range of sgp130-Fc	40–5000 ng/mL

**Table 2:** Intra- and inter-assay variation of CHO/SIE-Luc-based reporter assay.

IC_50_, ng/mL	Day 1	Day 2	Day 3	Mean	Inter-Assay CV (%)
Plate 1	487	463	505	485	4.3
Plate 2	501	495	491	496	1.0
Plate 3	494	484	486	488	1.1
Mean	494	481	494	/	/
Intra-assay CV (%)	1.2	3.4	2.0	/	/

**Table 3:** Recovery rates of sgp130-Fc samples by reporter assay.

Test No.	Relative Potency of Test Sample (%)	Relative Potency of Recovery Sample (%)	Recovery Rate
1	116.7	111.3	105.9%
2	106.2	106.2	106.2%
3	109.9	102	94.1%
4	98.3	99.4	100.6%
5	99.1	102	104.9%
6	100.9	102.9	104.9%
Mean	105.2	104.0	102.8%
Inter-assay CV	6.8%	4.0%	4.6%

**Table 4:** Copy numbers of luciferase and GAPDH in CHO/SIE-Luc cells at different passages by digital PCR.

Passage of Cells	Luciferase (Copies/ug Genome)			GAPDH (Copies/ug Genome)			Luciferase Copies per Copy GAPDH
	1	2	Mean	1	2	Mean	
P10	44.99	44.09	44.54	481.92	473.67	477.79	0.093
P30	52.83	52.98	52.91	557.69	549.51	553.60	0.096
P60	45.95	46.99	46.47	488.39	479.62	484.00	0.095
